# The role of IL-6 in pathogenesis of abdominal aortic aneurysm in mice

**DOI:** 10.1371/journal.pone.0185923

**Published:** 2017-10-05

**Authors:** Michihide Nishihara, Hiroki Aoki, Satoko Ohno, Aya Furusho, Saki Hirakata, Norifumi Nishida, Sohei Ito, Makiko Hayashi, Tsutomu Imaizumi, Yoshihiro Fukumoto

**Affiliations:** 1 Division of Cardiovascular Medicine, Department of Internal Medicine, Kurume University School of Medicine, Kurume, Japan; 2 Cardiovascular Research Institute, Kurume University, Kurume, Japan; 3 International University of Health and Welfare, Fukuoka, Japan; Brigham and Women's Hospital, Harvard Medical School, UNITED STATES

## Abstract

Although the pathogenesis of abdominal aortic aneurysm (AAA) remains unclear, evidence is accumulating to support a central role for inflammation. Inflammatory responses are coordinated by various soluble cytokines of which IL-6 is one of the major proinflammatory cytokines. In this study we examined the role of IL-6 in the pathogenesis of experimental AAA induced by a periaortic exposure to CaCl_2_ in mice. We now report that the administration of MR16-1, a neutralizing monoclonal antibody specific for the mouse IL-6 receptor, mildly suppressed the development of AAA. The inhibition of IL-6 signaling provoked by MR16-1 also resulted in a suppression of Stat3 activity. Conversely, no significant changes in either NFκB activity, Jnk activity or the expression of matrix metalloproteinases (Mmp) -2 and -9 were identified. Transcriptome analyses revealed that MR16-1-sensitive genes encode chemokines and their receptors, as well as factors that regulate vascular permeability and cell migration. Imaging cytometric analyses then consistently demonstrated reduced cellular infiltration for MR16-1-treated AAA. These results suggest that IL-6 plays an important but limited role in AAA pathogenesis, and primarily regulates cell migration and infiltration. These data would also suggest that IL-6 activity may play an important role in scenarios of continuous cellular infiltration, possibly including human AAA.

## Introduction

Abdominal aortic aorta (AAA) is a local expansion of the aortic diameter due to a weakened aortic wall [1]. The absence of a precise mechanism for this pathology has hampered the development of effective therapeutic strategies. Consequently, surgical intervention (with a graft) is currently the only treatment option. Recent studies have highlighted the importance of tissue destructive inflammation in AAA pathogenesis [2–4]. Indeed, we and others have demonstrated that pharmacologic intervention against mediators of pro-inflammatory signaling, including c-Jun N-terminal kinase (Jnk) [5] and nuclear factor kappa B (NFκB) [6] are effective in suppressing tissue destruction in a mouse model of AAA. Further, there is now an accumulating body of evidence to support the idea that regulating inflammation is a promising strategy with which to control the progression of AAA [7].

While inflammation is an essential defense mechanism in allowing an organism to combat tissue damage and exogenous pathogens, inflammation can be harmful when it fails to self-limit, as exemplified by AAA. In addition to AAA, autoimmune diseases such as rheumatoid arthritis are also caused by non self-limiting inflammation. Recently, a series of biological agents have been introduced into clinical practice that target proinflammatory cytokines in autoimmune disease and dramatically improve clinical outcomes [8]. These biological agents, that include antibodies and decoy receptors for TNFα, IL-1β, and IL-6, are effective in suppressing otherwise uncontrolled inflammation in these diseases.

The improvement in clinical outcome provoked by inhibiting proinflammatory cytokines in autoimmune disorders suggests that this strategy may also prove to be effective in controlling inflammation provoked in AAA. Indeed, several reports have shown that the inhibition of TNFα [9, 10] or IL-1β [11] are effective in suppressing AAA development in animal models. IL-6 has also been implicated in the molecular circuitry for vascular inflammation in aortic diseases, including aortic dissection and AAA [12].

Despite an abundance of IL-6 in AAA tissue, exactly how IL-6 participates in AAA pathogenesis, and whether its suppression would be of benefit in controlling inflammation remains unknown [13]. We therefore investigated the effects of MR16-1, a rat monoclonal antibody specific for the mouse IL-6 receptor [14], in a murine model of AAA. Our findings indicate that despite a suppressed development of AAA, MR16-1's effects are modest. MR16-1 suppressed gene expression for chemokines, their receptors, and the peptidases that control vascular permeability and cellular infiltration. These effects were all achieved in the absence of a major impact on tissue degrading matrix metalloproteinases. Therefore, IL-6 would appear to play a limited role in AAA development, and primarily regulates cellular infiltration.

## Materials and methods

### Mouse model of AAA

All animal experimental protocols were approved by the Animal Experiments Review Boards of Kurume University. The mouse AAA model was created in male C57BL/6J mice (Charles River Laboratories Japan) at the age of 10–12 weeks by periaortic application of 0.5 M CaCl_2_, as described previously [5, 15]. Briefly, the mouse infrarenal aorta was exposed by laparotomy under general anesthesia with 2% isoflurane. Exposure to CaCl_2_ was achieved using small pieces of cotton soaked in 0.5 M CaCl_2_ for 20 minutes. We also performed sham operation with the exposure to normal saline instead of CaCl_2_, which served as a negative control for CaCl_2_ exposure. We performed experiments either with 1 week of observational period mainly to assess the short term response of inflammatory signaling, or with 6 weeks of observational period mainly to assess the morphology of AAA. One or 6 weeks after CaCl_2_ exposure, the mice were euthanized by an overdose of pentobarbital and tissue and blood samples harvested. The aorta was immediately excised to obtain RNA or protein samples. Excision for morphometric and histological analyses was after perfusion fixation at physiological pressure with 4% paraformaldehyde. We assessed AAA diameter by measuring the maximal diameter of infrarenal aorta. We also took the reference diameter of the abdominal aorta between the right and left renal arteries, which was not exposed to CaCl_2_ to calculate the ratio of AAA diameter to left suprarenal aorta.

### IL-6 receptor neutralizing antibody

MR16-1, a rat monoclonal antibody raised against the mouse IL-6 receptor, was provided by Chugai Pharmaceutical Co. Ltd; MR16-1 administration was used to neutralize the function of IL-6 [14]. We administered 2 mg MR16-1 by tail vein injection one day before exposure to CaCl_2_ to suppress IL-6 signaling and to induce tolerance to the rat antibody [16]. For 6-week experiments, the initial tail vein injection before CaCl_2_ exposure was followed by intraperitoneal injection of 0.25 mg MR16-1 every week to ensure the suppression of IL-6 effect throughout the observational period. For 1-week experiments, only tail vein injection of 2 mg MR16-1 was performed. Administration of physiological saline (NaCl) and non-specific rat IgG (MP Biomedicals #0855951) served as negative controls to MR16-1.

### Histology, immunostaining, and imaging cytometric analyses

Murine aortae were embedded in paraffin and 5 μm sections prepared for staining. Hematoxylin and eosin (H&E), elastica van Gieson (EVG) or Von Kossa stains were performed. Immunohistochemical staining of aortic tissue was performed for macrophages (Iba1; Wako Pure Chemical Industries #019–19741) and Mcp-1 (Abcam #ab25124). The area of Von Kossa or Mcp-1 staining and the tissue area was measured by BZ Analyzer software (Keyence).

We performed imaging cytometric analyses for aortic samples with immunofluorescence staining using ArrayScan XTI (ThermoFisher Scientific) and the FlowJo Software (FlowJo LLC). Briefly, tissue sections from four mouse aortae in each experimental group were processed for 3-color fluorescence staining; DAPI for nuclei, smooth muscle α-actin (SMA) antibody (clone 1A4, Sigma-Aldrich #A2547) for smooth muscle cells, and antibodies specific for either phospho-Stat3 (clone D3A7, Cell Signaling Technology #9145), phospho-Smad2 (clone 138D4, Cell Signaling Technology #3108), or NFκB (clone D14E12, Cell Signaling Technology #8242). Staining conditions were determined by single staining with a given antibody and validated to show identical results in 3-color staining. All samples in a given set of analysis were processed with the same staining condition at the same time and photomicrographs were taken with the same exposure condition to obtain consistent results. In the imaging cytometric analyses with ArrayScan XTI, each cell was identified by nuclear DAPI staining and arbitrarily gated to separate the positive (smooth muscle cells) and negative (non-smooth muscle cells) populations for SMA signal surrounding the nucleus. The gates for the nuclear signal intensity of phospho-Stat3, phospho-Smad2 and NFκB were also determined arbitrarily. These gates were set constant throughout the experimental groups in a given set of analysis.

### Immunoblotting and gelatin zymography

Immunoblots for Jnk (rabbit polyclonal antibody, Abcam), phospho-Jnk (clone 98F2, Cell Signaling Technology), Stat3 (rabbit polyclonal antibody, Cell Signaling Technology), phospho-Stat3 (clone D3A7, Cell Signaling Technology), and Lox (rabbit polyclonal antibody, Abcam) were performed using the NuPAGE system (ThermoFisher Scientific). Gelatin zymography was performed using the Novex zymogram system (ThermoFisher Scientific) according to the manufacturer's instruction. Serum cytokine concentrations were quantified using a Bio-Plex multiplex bead array system with the mouse cytokine Th17 panel A (#M6000007NY, Bio-Rad).

### Transcriptome analysis

The aortic tissue was snap-frozen in liquid nitrogen immediately after killing the mice and obtaining the tissue. The aortic tissue was homogenized in TRIzol reagent (Thermofisher Scientific), and total RNA was isolated using RNeasy kit (Qiagen) according to the instruction by the manufacturer. Transcriptome analyses were performed using a SurePrint G3 Mouse GE microarray 8x60K (Agilent Technologies). Functional annotation clusters were obtained using the Database for Annotation, Visualization, and Integrated Discovery (DAVID, https://david.ncifcrf.gov/) [17] with the Gene Ontology terms set to GOTERM_BP_FAT, GOTERM_CC_FAT and GOTERM_MF_FAT.

### Statistical analyses

Statistical analyses were performed using GraphPad PRISM 5 (GraphPad Software). One way ANOVA was performed to compare 3 or more groups followed by Bonferroni's multiple comparison test, when the data passed D'Agostino and Pearson normality test and Bartlett's test for equal variances. Otherwise, Kruskal-Wallis test was performed followed by Dunn's multiple comparison test. Statistical significance was indicated by a p value of less than 0.05.

## Results

### The effect of IL-6 receptor neutralization on the development of AAA

**Fig 1:**
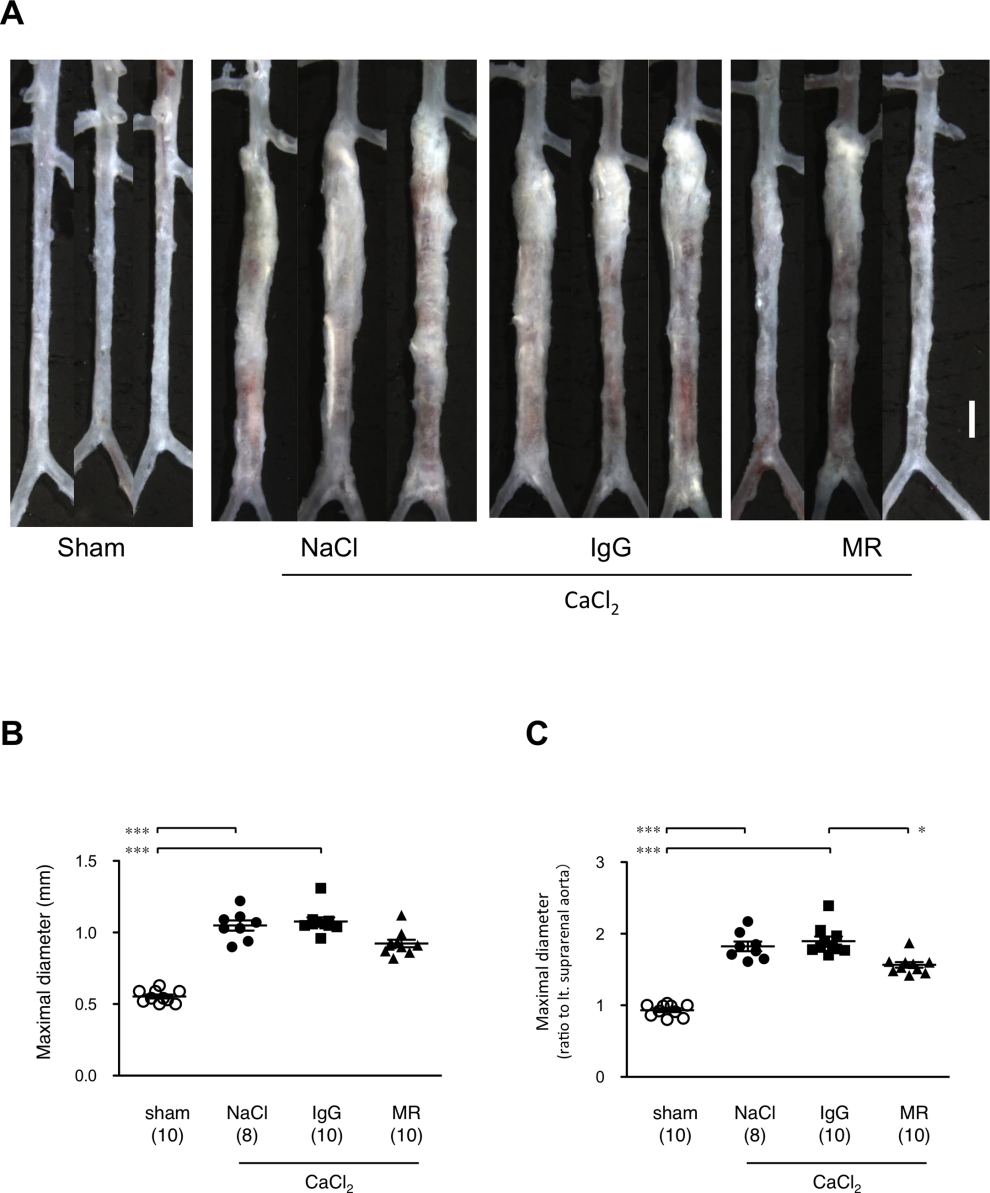
The effect of MR16-1 on the development of AAA. (A) Morphometric analyses of mouse AAA, (B) maximal aortic diameters in millimeters, and (C) relative to an internal reference diameter at the level of the left renal artery. In panel A, CaCl_2_ indicates the AAA model 6 weeks after CaCl_2_ exposure. NaCl and IgG indicate the AAA model treated with physiological saline and non-specific rat IgG, respectively, as negative controls for MR16-1 (denoted MR). The white bar denotes 1 mm. In panels B and C, symbols indicate individual data and bars indicate means ± standard errors. The numbers of mice for observation are indicated in parenthesis. *p< 0.05 and ***p<0.001.

We used a well-established mouse model of AAA that employs a periaortic application of 0.5 M CaCl_2_ for 20 min (CaCl_2_ exposure) to the exposed aorta [5, 15, 18]. This procedure induces chronic inflammation of the aortic wall that results in tissue destruction and the development of AAA. As shown in Fig 1, CaCl_2_ exposure provoked an increase in the maximum diameter of the aorta from 0.55 ± 0.01 mm to 1.05 ± 0.04 mm, 6 weeks after CaCl_2_ exposure. We then administered the MR16-1 reagent to mice, which is a rat monoclonal antibody directed against the mouse IL-6 receptor, in order to neutralize the function of IL-6 [14]. As shown in Fig 1B, administration of MR16-1 resulted in a significant reduction in the maximal aortic diameter 6 weeks after CaCl_2_ exposure (0.92 ± 0.03 mm). On the other hand, a control rat IgG or saline injection failed to alter the maximal aortic diameter in the same time frame (i.e. 6 weeks after the CaCl_2_ exposure) (1.08 ± 0.03 mm). When we compared the maximal diameter to the aortic diameter just above the left renal artery as an internal reference, the data were essentially identical (Fig 1C). These results indicate that in this mouse model of AAA, IL-6 signaling is required for the full development of AAA.

### Microscopic findings in the AAA model

**Fig 2:**
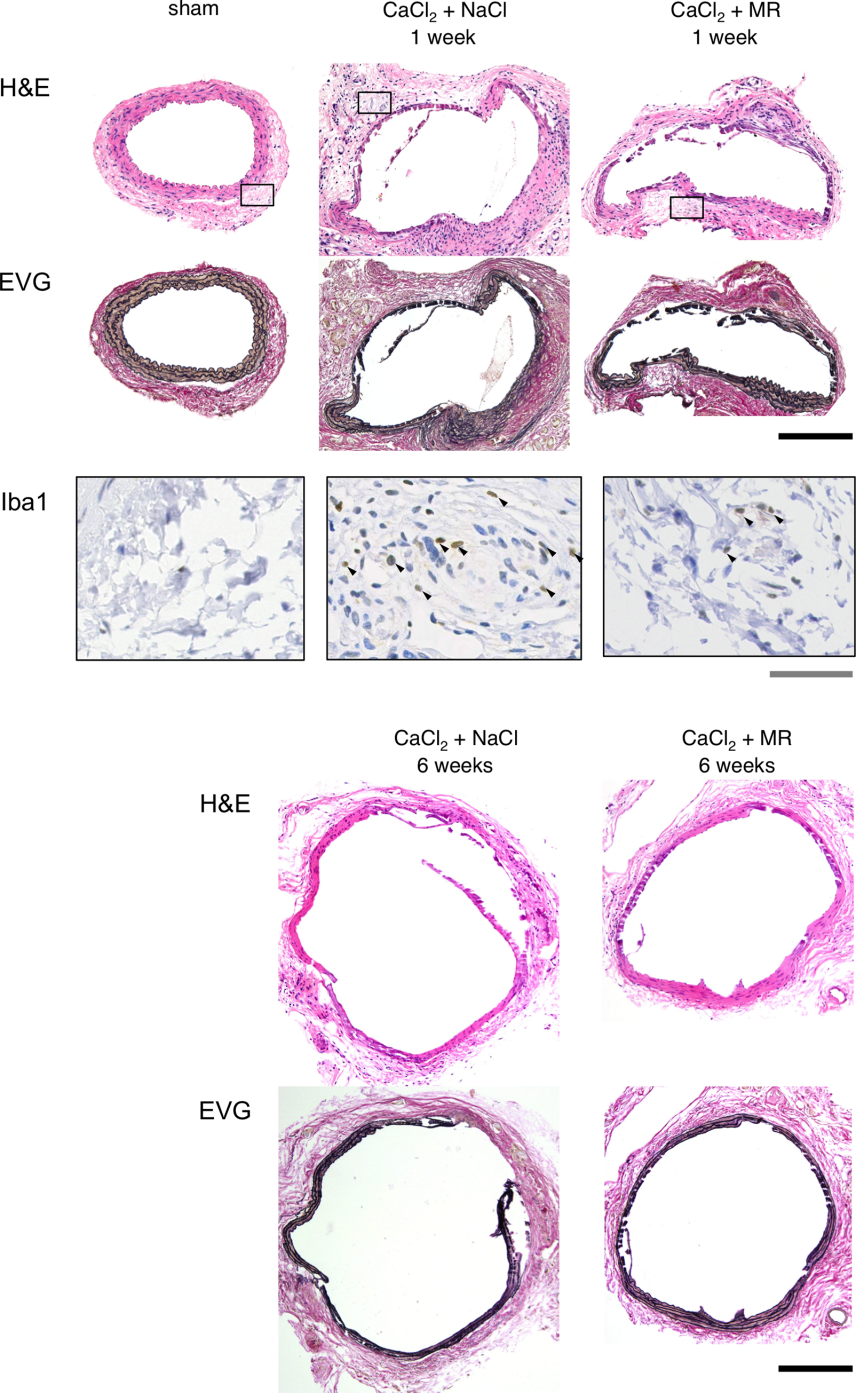
Histological study of AAA. Histological findings are shown for perfusion-fixed aortae at their maximum diameter by hematoxylin-eosin (H&E), and elastica van Gieson (EVG) staining. Tissue samples were obtained prior to, 1 week and 6 weeks after CaCl_2_ exposure, with or without MR16-1 administration. Immunohistochemical staining of Iba1, a marker for monocyte/macrophages, was also performed 1 week after CaCl_2_ exposure. Rectangles in H&E staining indicate the area for Iba1 staining. The black and gray bars denote 200 μm and 5 μm, respectively. Arrowheads indicate Iba1-positive cells.

Histological examination revealed prominent degeneration of the medial layers with substantial cellular infiltration in the adventitial layers of the aortic wall 1 week after CaCl_2_ exposure (Fig 2). Immunohistochemical stainings demonstrated abundant infiltration of cells that were positive for Iba1, a marker of monocytes/macrophages into the aortic tissue at this time point. Aortic tissue with MR16-1 administration showed less amount of Iba1 positive cells. Six weeks after CaCl_2_ exposure, medial degeneration was further progressed with flattened and elongated elastic lamellae. At this time point, the adventitial layer demonstrated less cellular infiltration compared to that observed 1 week after CaCl_2_ exposure. The administration of MR16-1 was found to modestly suppress adventitial cellular infiltration at 1 week and medial degeneration 6 weeks after CaCl_2_ exposure, although suppression was incomplete. These findings are consistent with the modest decrease in AAA diameter provoked by MR16-1 as demonstrated macroscopically.

**Fig 3:**
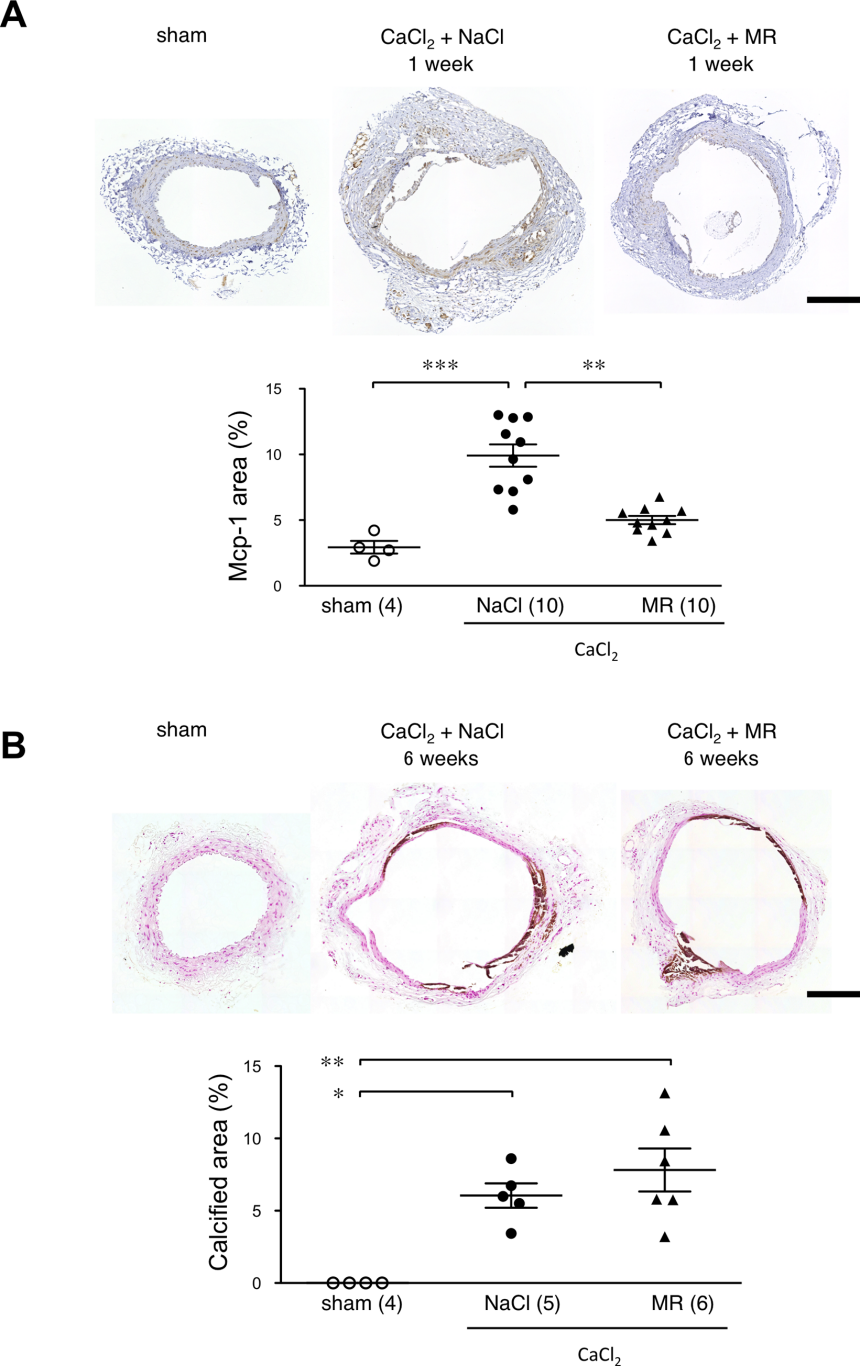
Effect of MR16-1 on Mcp-1 expression and calcification. (A) Photomicrographs are shown for Mcp-1 staining of aortic tissue with sham operation, 1 week after CaCl_2_ exposure with saline treatment (CaCl_2_+NaCl), and 1 week after CaCl_2_ exposure with MR16-1 treatment (CaCl_2_+MR). Expression of Mcp-1 was evaluated by calculating the ratio of Mcp-1-positive area to the tissue area (Mcp-1 area). (B) Von Kossa staining was performed for aortic tissue with sham operation (sham), 6 weeks after CaCl_2_ exposure with saline treatment (CaCl_2_+NaCl), and 6 weeks after CaCl_2_ exposure with MR16-1 treatment (CaCl_2_+MR). The extent of aortic tissue calcification was evaluated by calculating the ratio of the calcified (brown) area to the tissue area (Calcified area). Symbols in graphs indicate individual data and bars indicate means ± standard errors. The numbers of mice for observation are indicated in parentheses. *p<0.05, **p<0.01, and ***p<0.001. Bars denote 200 μm.

As we observed infiltration of monocytes/macrophages in AAA tissue, we examined the expression of monocyte chemoattractant protein-1 (Mcp-1) that has been reported to participate in AAA pathogenesis [19, 20] by immunohistochemistry (Fig 3A). At the baseline, we observed weak Mcp-1 staining in the medial layer of the aorta. One week after the CaCl_2_ exposure followed by the saline injection, Mcp-1 staining became more prominent in the media and adventitia of the aortic tissue On the other hand, administration of MR16-1 significantly suppressed the Mcp-1 positive area in the aortic tissue, indicating that IL-6 was responsible at least in part for Mcp-1 expression in the aortic tissue.

Because calcification is a common feature of the CaCl_2_-induced AAA model and human AAA [4], and IL-6 may be causally related to vascular calcification [21], we also evaluated the effect of MR16-1 on the extent of aortic wall calcification (Fig 3B). Von Kossa staining showed no calcification of the aortic wall at the baseline, but prominent aortic wall calcification 6 weeks after the CaCl_2_ exposure. Administration of MR16-1 did not cause significant changes in the ratio of the calcified area to the tissue area, suggesting that IL-6 may not play a major role in aortic calcification in this experimental setting.

### Serum cytokine profiling in AAA model

**Fig 4:**
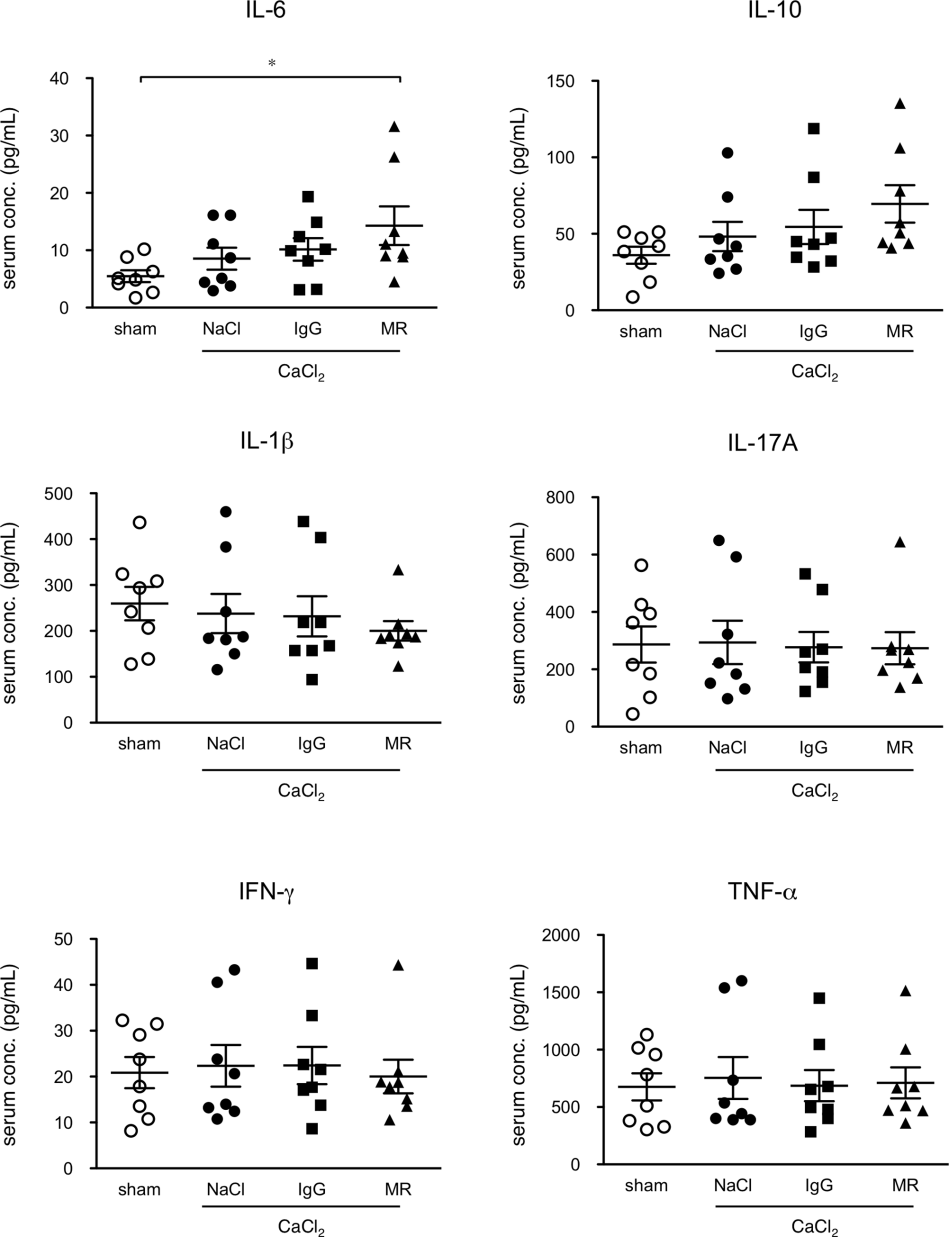
Effect of MR16-1 on serum cytokine profiles. Serum concentrations of cytokines are shown for mice without (sham) and with AAA, treated with physiological saline (NaCl), non-specific rat IgG (IgG), and MR16-1 (MR), measured 1 week after CaCl_2_ exposure. Symbols indicate individual data and bars indicate means ± standard errors from 8 independent observations in each experimental group. *p<0.05.

We examined the serum cytokine profiles of mice 1 week after CaCl_2_ exposure (Fig 4), when the inflammatory response is at its peak [5]. Following CaCl_2_ exposure with physiological saline (NaCl) injection or control rat IgG injection, none of the cytokines showed significant changes, which is consistent with our previous report of CaCl_2_ exposure not provoking a systemic cytokine response [22]. Administration of MR16-1 resulted in a significant increase compared to sham-operated group in the serum concentration of IL-6, which most likely reflects a release of receptor-bound IL-6 by MR16-1, as previously reported [23]. This effect is compatible with the notion of MR16-1 inhibiting IL-6 binding to its receptor.

### Inflammatory signaling in AAA tissue

**Fig 5:**
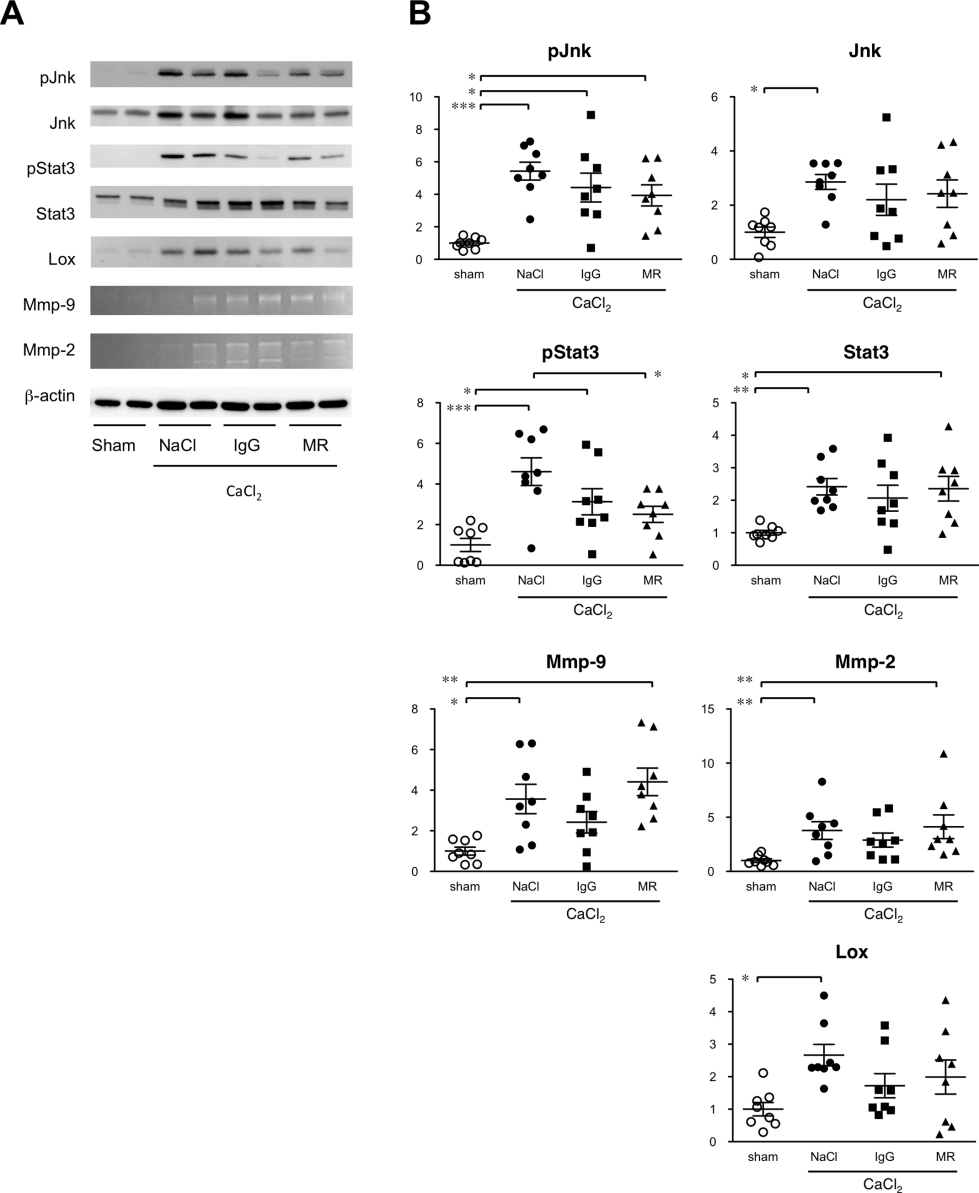
Effect of MR16-1 on inflammatory signaling in AAA. Molecular activities of inflammatory signaling molecules in aortic tissue. (A) Immunoblots for phospho-Jnk (P-Jnk), total Jnk, phospho-Stat3 (pStat3), total Stat3, and lysyl oxidase are shown. Gelatin zymograms are also shown for Mmp-9 and Mmp-2. Expression of β-actin served as an internal loading control. (B) Quantitative analysis of immunoblots and gelatin zymograms are shown in which a sham-operated aorta was assigned a value of 1. Symbols indicate individual data and bars indicate means ± standard errors from 8 independent observations in each experimental group. *p<0.05, **p<0.01, and ***p<0.001.

The fundamental mechanism of AAA appears to involve an inflammatory response that promotes tissue degradation. We therefore examined the activation status of Jnk, a critical mediator of AAA pathogenesis [5] and Stat3, a well-established mediator of IL-6 signaling by immunoblot analysis 1 week after CaCl_2_ exposure (Fig 5). CaCl_2_ exposure caused significant increases in the protein expressions of Jnk and Stat3 and their activities. Although administration of MR16-1 seemed to result in less significant activation of Jnk, the effect was similar to control rat IgG. On the other hand, MR16-1, but not control rat IgG, diminished Stat3 activation by CaCl_2_ exposure. We next examined effectors that are involved in the metabolism of the extracellular matrix (ECM). CaCl_2_ treatment caused significant increases in Mmp-9 and Mmp-2 expressions, as shown by the gelatin zymography, and lysyl oxidase (Lox) as shown by immunoblots of aortic samples. The administration of MR16-1 provoked no significant change in the levels of Mmp-9, Mmp-2, or Lox. These results were consistent with the notion that Stat3 is the main mediator of IL-6 signaling. Suppressive effect of MR16-1 on AAA expansion did not seem to involve the changes in Jnk activity or ECM metabolic enzymes as examined in this study.

### Signaling at the cellular level in AAA tissue

**Fig 6:**
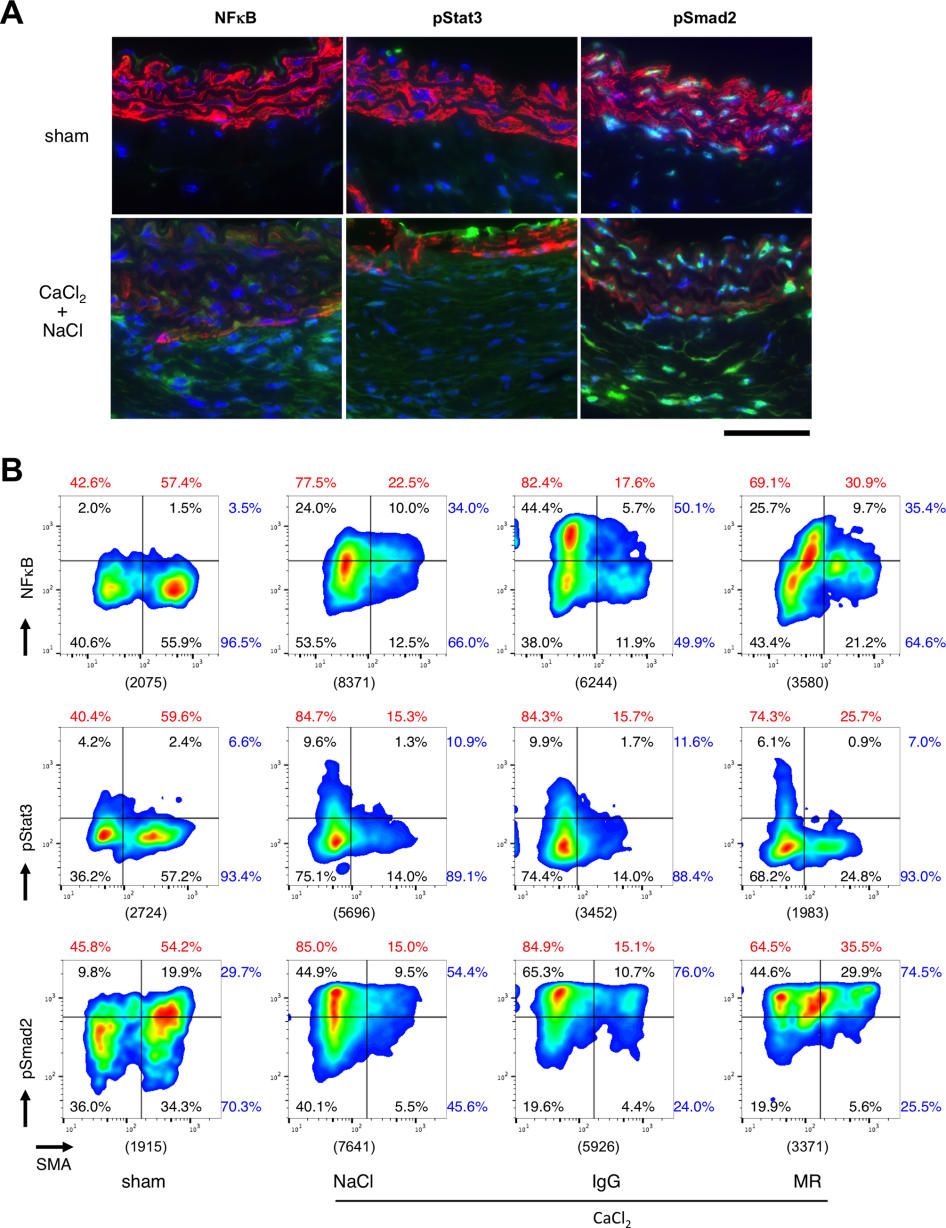
Effect of MR16-1 on cellular signaling. (A) Representative photomicrographs are shown for 3-color immunofluorescence staining of aortic tissue without (sham) or with CaCl_2_ exposure followed by saline injection (NaCl) or control rat IgG injection (IgG). Blue and red colors indicate nuclear and SMA stainings, respectively. Green color indicate NFκB, pStat3 or pSmad2. All samples are shown with the luminal side up. A bar denote 50 μm. (B) Scattergrams of imaging cytometric analyses are shown for nuclear signals of NFkB, pStat3 and pSmad2, together with cytosolic signal of SMA. Cell percentages for each quadrant are shown in black. The percentages of active vs. inactive NFκB, pStat3 and pSmad2 are indicated by blue numbers. The percentages of SMA-negative and -positive populations are shown in red numbers. The counts of total cells are shown in parenthesis below each panel. Data were obtained from 4 mouse aortae in each experimental group.

To better understand the role of IL-6 signaling in AAA pathogenesis at the cellular level, we examined the nuclear localization of NFκB, a mediator of AAA pathogenesis [6], pStat3, a mediator of IL-6 signaling, and pSmad2, a mediator of TGFβ signaling that is involved in inflammation, ECM metabolism, and regulation of smooth muscle cell function [24], together with staining for smooth muscle α-actin (SMA), a marker of smooth muscle cells (SMCs) (Fig 6A). In aorta from sham-operated mice, nuclear localization of NFκB and pStat3 were negligible, indicative of the absence of their activities at the baseline. On the other hand, pSmad2 showed strong signal mainly in medial SMCs. CaCl_2_ exposure resulted in the increase in adventitial cells with nuclear NFκB, concomitant with the decrease in SMA staining. It also caused increase in pStat3 in part of adventitial and intimal cells, increase in nuclear pSmad2 in adventitia, but decrease in nuclear pSmad2 in medial cells.

We performed imaging cytometric analysis of these aortic tissue (Fig 6B). When gates were set to arbitrarily separate the SMA-positive and -negative populations, approximately 55% of the total cell population was SMA-positive, and approximately 45% SMA-negative, presumably indicating SMCs and non-SMCs, respectively, in NFκB, pStat3 and pSmad2 analyses. CaCl_2_ treatment caused a dramatic increase in non-SMCs to approximately 80% (Fig 6B). Administration of MR16-1 resulted in the decrease in SMA-negative population to 70% and increase in SMA-positive population to 30%, whereas control rat IgG did not show such an effect. CaCl_2_ exposure caused an increase in nuclear NFκB of both SMA-negative and -positive populations from 3.5% to 34% of total population, for which MR16-1 did not show discernible effect. CaCl_2_ exposure also caused an increase in nuclear pStat3 from 6.6% to 10.9% mainly in SMA-negative population, which was reduced by MR16-1 administration to 7% whereas control rat IgG showed no effect. CaCl_2_ exposure caused increase in pSmad2 mainly in SMA-negative population and reduction in SMA-pSmad2 double positive population from 19.9% to 9.5%. MR16-1 administration provoked a recovery of the SMA-pSmad2 double positive population to 29.9%.

These results suggested that CaCl_2_ exposure caused the reduction of SMA-positive population possibly due to the cellular infiltration, reduction in SMA expression in SMCs or loss of SMCs, which was associated with the activation of NFkB, Stat3 and Smad2 mainly in SMA-negative population. MR16-1 caused a partial recovery of SMA-positive population, which was associated with the decrease in Stat3 activity but not with the changes in NFκB activity.

### Transcriptome analysis during AAA development

To better understand the role of IL-6 in the pathogenesis of AAA, we performed a transcriptome analysis 1 week after exposure to CaCl_2_, with or without MR16-1 administration. Functional annotation clusters were obtained using the Database for Annotation, Visualization, and Integrated Discovery (DAVID). Of the 55,681 probes on the DNA microarray, 5,940 and 4,694 probes with matched DAVID IDs were up- and down-regulated, respectively, by CaCl_2_ treatment, as defined by a fold change of more than 1.5x and p<0.05 for up-regulated probes, or less than 0.67x and p<0.05 for down-regulated probes (Table 1). Of the genes with CaCl_2_-induced changes, only 3.1% were down-regulated, and 2.2% up-regulated by MR16-1.

**Table 1:** Effect of MR16-1 on changes in gene expression.

	CaCl_2_ / Pre		
CaCl_2_ + MR / CaCl_2_	Up	Down	Total
No change	5,520 (51.9%)	4,546 (42.7%)	10,066
Down	300 (2.8%)	34 (0.3%)	334
Up	120 (1.1%)	114 (1.1%)	234
Total	5,940	4,694	10,634

The effects of CaCl_2_ exposure and MR16-1 treatment on gene expression profiles. Changes in gene expression are indicated by ratios with and without CaCl_2_ treatment. Similarly, antibody effects are shown by changes in gene expression ratios with MR16-1 (i.e. CaCl_2_ + MR) and without MR16-1 treatment (CaCl_2_ only). Expression changes of at least 1.5 fold (UP) or 0.67 fold (DOWN), with p<0.05, are defined as significant. The numbers in parentheses indicate the percentages in the DNA microarray.

We focused on the genes that were induced by CaCl_2_ and suppressed by MR16-1. The annotation cluster with the highest enrichment score (8.59) revealed GO terms that included immune response, chemotaxis, and inflammatory response (Table 2). Most of the genes in this annotation cluster were for chemokines, Fc receptors, and interleukins (Table 3). The second annotation cluster (enrichment score 2.57) showed GO terms for peptidase and hydrolase activities. The genes in this annotation cluster were for coagulation factors, kallikreins, and their related peptidases that work in concert to increase vascular permeability and cell migration. The third annotation cluster (enrichment score 2.46) showed GO terms for G-protein coupled receptors. Many of the genes in this annotation cluster were for chemokine receptors. Therefore, the transcriptome and the functional annotation analyses suggested that IL-6 promotes cell migration and infiltration during the development of AAA.

**Table 2:** Annotation clusters affected by MR16-1.

**Annotation Cluster 1**, Enrichment Score: 8.59		
Term	Count	P value
immune response	20	5.28E-10
chemotaxis	14	1.29E-09
inflammatory response	20	2.51E-08
**Annotation Cluster 2**, Enrichment Score: 2.57		
Term	Count	P Value
serine-type endopeptidase activity	14	2.36E-06
serine-type peptidase activity	11	3.51E-05
hydrolase activity, acting on carbon-nitrogen (but not peptide) bonds, in linear amides	5	3.15E-04
proteolysis	16	0.003686374
endopeptidase activity	6	0.012566252
peptidase activity	13	0.026610026
hydrolase activity	25	0.133226224
protein complex	8	0.657526617
**Annotation Cluster 3**, Enrichment Score: 2.46		
Term	Count	P Value
G-protein coupled receptor signaling pathway	37	3.74E-04
signal transducer activity	19	0.001144267
G-protein coupled receptor activity	33	0.014962721
signal transduction	24	0.022169421

Annotation clusters with enrichment scores in excess of 2.0 are shown for MR16-1-sensitive genes.

**Table 3:** Genes in annotation clusters 1, 2, and 3.

**Annotation Cluster 1, Enrichment Score: 8.59**	matrix metallopeptidase 1b (interstitial collagenase)(Mmp1b)
2'-5' oligoadenylate synthetase-like 1(Oasl1)	pancreatic lipase(Pnlip)
Fc receptor, IgA, IgM, high affinity(Fcamr)	peroxiredoxin 6(Prdx6)
Fc receptor, IgG, low affinity IIb(Fcgr2b)	phospholipase A2, group X(Pla2g10)
Mediterranean fever(Mefv)	protease, serine 34(Prss34)
chemokine (C-C motif) ligand 12(Ccl12)	protease, serine 38(Prss38)
chemokine (C-C motif) ligand 7(Ccl7)	protein tyrosine phosphatase, non-receptor type 5(Ptpn5)
chemokine (C-C motif) ligand 8(Ccl8)	ring finger protein 165(Rnf165)
chemokine (C-C motif) receptor 1-like 1(Ccr1l1)	transient receptor potential cation channel, subfamily C, member 4(Trpc4)
chemokine (C-C motif) receptor 2(Ccr2)	trypsin 4(Try4)
chemokine (C-C motif) receptor 3(Ccr3)	trypsin 5(Try5)
chemokine (C-C motif) receptor 5(Ccr5)	
chemokine (C-C motif) receptor 9(Ccr9)	**Annotation Cluster 3, Enrichment Score: 2.46**
chemokine (C-X-C motif) ligand 10(Cxcl10)	5-hydroxytryptamine (serotonin) receptor 1D(Htr1d)
chemokine (C-X-C motif) ligand 13(Cxcl13)	5-hydroxytryptamine (serotonin) receptor 2C(Htr2c)
chemokine (C-X-C motif) receptor 1(Cxcr1)	5-hydroxytryptamine (serotonin) receptor 7(Htr7)
chitinase-like 1(Chil1)	EDAR-associated death domain(Edaradd)
chitinase-like 3(Chil3)	G protein-coupled receptor 182(Gpr182)
complement component 6(C6)	G protein-coupled receptor 35(Gpr35)
cysteinyl leukotriene receptor 1(Cysltr1)	G protein-coupled receptor 82(Gpr82)
cytotoxic T-lymphocyte-associated protein 4(Ctla4)	G protein-coupled receptor, family C, group 5, member B(Gprc5b)
formyl peptide receptor 1(Fpr1)	SRY (sex determining region Y)-box 8(Sox8)
formyl peptide receptor, related sequence 4(Fpr-rs4)	adenosine A2a receptor(Adora2a)
free fatty acid receptor 2(Ffar2)	chemokine (C-C motif) ligand 12(Ccl12)
granzyme D(Gzmd)	chemokine (C-C motif) ligand 7(Ccl7)
interferon activated gene 202B(Ifi202b)	chemokine (C-C motif) ligand 8(Ccl8)
interleukin 1 alpha(Il1a)	chemokine (C-C motif) receptor 1-like 1(Ccr1l1)
interleukin 1 beta(Il1b)	chemokine (C-C motif) receptor 2(Ccr2)
interleukin 10(Il10)	chemokine (C-C motif) receptor 3(Ccr3)
interleukin 2 receptor, alpha chain(Il2ra)	chemokine (C-C motif) receptor 5(Ccr5)
lymphotoxin B(Ltb)	chemokine (C-C motif) receptor 9(Ccr9)
roundabout guidance receptor 2(Robo2)	chemokine (C-X-C motif) ligand 10(Cxcl10)
	chemokine (C-X-C motif) ligand 13(Cxcl13)
**Annotation Cluster 2, Enrichment Score: 2.57**	chemokine (C-X-C motif) receptor 1(Cxcr1)
CD38 antigen(Cd38)	cysteinyl leukotriene receptor 1(Cysltr1)
DEAD (Asp-Glu-Ala-Asp) box polypeptide 4(Ddx4)	cysteinyl leukotriene receptor 2(Cysltr2)
RIKEN cDNA 4933425L06 gene(4933425L06Rik)	formyl peptide receptor 1(Fpr1)
arginase, liver(Arg1)	formyl peptide receptor, related sequence 4(Fpr-rs4)
bone marrow stromal cell antigen 1(Bst1)	free fatty acid receptor 2(Ffar2)
cathepsin C(Ctsc)	olfactory receptor 107(Olfr107)
cell migration inducing protein, hyaluronan binding(Cemip)	olfactory receptor 1122(Olfr1122)
chitinase-like 3(Chil3)	olfactory receptor 1320(Olfr1320)
coagulation factor VII(F7)	olfactory receptor 1412(Olfr1412)
coagulation factor X(F10)	olfactory receptor 1496(Olfr1496)
dipeptidase 3(Dpep3)	olfactory receptor 294(Olfr294)
epidermal growth factor binding protein type B(Egfbp2)	olfactory receptor 358(Olfr358)
excision repair cross-complementing rodent repair deficiency, complementation group 6 like 2(Ercc6l2)	olfactory receptor 591(Olfr591)
family with sequence similarity 186, member B(Fam186b)	olfactory receptor 749(Olfr749)
granzyme D(Gzmd)	olfactory receptor 859(Olfr859)
kallikrein 1(Klk1)	olfactory receptor 873(Olfr873)
kallikrein 1-related peptidase b24(Klk1b24)	olfactory receptor 920(Olfr920)
kallikrein 1-related peptidase b27(Klk1b27)	olfactory receptor 969(Olfr969)
kallikrein 1-related peptidase b3(Klk1b3)	vomeronasal 2, receptor 102(Vmn2r102)
kallikrein 1-related petidase b26(Klk1b26)	wingless-type MMTV integration site family, member 4(Wnt4)

## Discussion

Recent advances in our understanding of the molecular pathogenesis of AAA have demonstrated that AAA is an ordered process of inflammation, changes to the cellular composition of the aortic wall, and ECM remodeling. These processes eventually lead to a local weakening of the aortic wall and its expansion [1]. Understanding AAA pathogenesis provides potential therapeutic opportunities at various levels, including extracellular mediators, cell surface receptors, intracellular signaling molecules, and regulators of gene expression [2–4]. Of these, extracellular mediators and their receptors are favorable therapeutic targets given their ease of modulation by pharmacologic agents. This was one of the reasons for our focus on IL-6, as IL-6 is one of the most abundantly expressed cytokines in AAA tissue [13].

Our findings demonstrate that inhibition of IL-6 can suppress Stat3 activation and the full expansion of AAA, indicating that IL-6 is a major cytokine in the tissue activation of Stat3. However, the suppression of AAA diameter was limited, indicating that IL-6 plays an important albeit partial role in the pathogenesis of AAA. An alternative interpretation is that effect of MR16-1, a rat IgG, was masked due to the immune response against rat IgG in mice as suggested previously [25], although we used a protocol to induce a tolerance in mice against rat IgG [16] and a number studies have shown the suppressive effect of MR16-1 on IL-6 signaling, including those for cardiovascular disease [26–28]. Indeed, MR16-1 administration in this study resulted in the increase in serum IL-6 indicative of its release from the endogenous receptor, and suppression of Stat3 activation. We therefore concluded that MR16-1 was effective in at least partly suppressing IL-6 signaling.

IL-6 inhibition by MR16-1 resulted in no discernible effect on Jnk or NFκB, critical mediators in AAA signaling [5, 6]. Consistently, IL-6 inhibition did not show a significant effect on the expressions of Mmp-2 or Mmp-9 that are involved in AAA pathogenesis [18]. A limited role for IL-6 was also demonstrated by transcriptome analysis, as we identified immune cell migration as an enriched function in IL-6-dependent genes. Consistent with the result of transcriptome analysis, expression of Mcp-1 was IL-6-dependent at the protein level, as demonstrated by immunohistochemical analysis of AAA tissue in this study. Imaging flow cytometric analysis showed that CaCl_2_ dramatically increased the non-VSMC fraction in aortic walls, probably due to the infiltration of inflammatory cells. This effect was partially suppressed by the administration of MR16-1. These findings are consistent with the notion that IL-6 is involved in the expression of chemokines and chemokine receptors that regulate cell migration [29, 30].

Inhibition of IL-6 showed no significant effect on Jnk or NFκB activities or the expressions of Mmp-2 and Mmp-9, indicating that IL-6 does not play a major role in tissue destructive inflammation during AAA. Promoter analyses of Mmp-9 have consistently revealed binding sites for NFκB, SP-1, and AP-1, but not for STAT [31, 32]. Conversely, it has been reported that Mmp-9 expression is inhibited by IFNβ and IFNγ in a STAT1-dependent manner [33]. IL-6 inhibition moderately suppressed AAA development, although it is unlikely to achieve full regression of AAA. Several mechanisms may be involved in the development of IL-6-dependent AAA. These may include the expression of tissue degrading proteases other than Mmp-2 or Mmp-9, the augmentation of inflammation by IL-6-mediated cell infiltration, and ECM remodeling. For example, it has been reported that IL-6 signaling significantly increases the expression of MMP-1, MMP-3, and MMP-13, but not MMP-9 [32], all of which are likely to be involved in AAA pathogenesis [34]. It has also been proposed that Stat3-activated fibroblasts may participate in lung fibrosis [35], which shares ECM deposition and remodeling as a common feature with AAA. Whether and how these mechanisms participate in IL-6 dependent AAA pathogenesis now warrants further investigation.

Our data using a mouse model indicate an important albeit limited role for IL-6 in AAA pathogenesis. However, human AAA is a more heterogeneous disease than the mouse model in terms of pathogenesis and the time course of the disease [3]. The human AAA wall in a single patient manifests various stages of disease including inflammation, destruction, and fibrosis of the tissue [1]. IL-6 may play different roles depending on pathogenesis and disease stage. Another important difference between mouse and human AAA is that the inflammation in mouse AAA is self-limiting, unlike the human scenario of continuous and progressive inflammation [30]. This non-limiting inflammation presumably requires a continuous influx of inflammatory cells from the blood stream. IL-6 may be important in such situations of continuous and progressive inflammation in order to promote cellular infiltration. IL-6 may also be important in the aortic wall inflammation triggered by the implantation of a stent-graft [36] during endovascular aneurysm repair (EVAR). If this is the case, intervening in IL-6 signaling may be of use in regulating excessive acute inflammation and in improving clinical outcome. In this regard, it is noteworthy that a systematic review and meta-analysis proposed that the IL-6 receptor pathway may be causally involved in human AAA pathogenesis [37]. Therefore, the role of IL-6 in human AAA warrants further investigation.

In conclusion, our data suggest that IL-6 plays an important but limited role in the pathogenesis of AAA, which appears to primarily involve cell migration and infiltration, with minor effects on tissue disruption during inflammation. Should these findings be applicable to human AAA, then the level of IL-6 in AAA tissue may reflect the degree of ongoing inflammatory cell flux. Further research is now needed to understand the exact role of IL-6 in order to establish improved diagnostic and therapeutic strategies for AAA.
